# Psychiatrists′ work with sickness certification: frequency, experiences and severity of the certification tasks in a national survey in Sweden

**DOI:** 10.1186/1472-6963-12-362

**Published:** 2012-10-17

**Authors:** Gunnar H Nilsson, Britt Arrelöv, Christina Lindholm, Therese Ljungquist, Linnea Kjeldgård, Kristina Alexanderson

**Affiliations:** 1 Department of Neurobiology, Care Sciences and Society, Division of Family Medicine, Karolinska Institutet, Stockholm, Sweden; 2 Stockholm County Council, Stockholm, Sweden; 3 Department of Clinical Neuroscience, Division of Insurance Medicine, Karolinska Institutet, Stockholm, Sweden; 4 Center for Family and Community Medicine, Karolinska Institutet, Alfred Nobels allé 12, SE-141 83, Huddinge, Sweden

**Keywords:** Sickness certification, Psychiatry, Sick leave, Physician

## Abstract

**Background:**

Many psychiatrists are involved in sickness certification of their patients; however, there is very limited knowledge about this aspect of their work. The objective of this study was to explore frequencies of problematic issues in the sickness certification tasks and experiences of severity regarding these problematic issues among psychiatrists.

**Methods:**

A cross-sectional nationwide questionnaire study to all physicians in Sweden. The 579 specialists in psychiatry who answered the questionnaire, were under 65 years of age, worked mainly in psychiatric care, and had consultations involving sickness certification at least once a week were included.

**Results:**

The frequency of problematic sickness certification consultations a few times per year or more often was considered by 87.3% of the psychiatrists; 11.7% handle such cases at least once a week. A majority (60.9%) reported ‘not having enough time with the patient’ at least once a week. The psychiatrists had access to several categories of professionals in their daily work. More than one third certified unnecessarily long sick-leave periods at least once a month due to waiting times for Social Insurance Office investigations or for treatments or investigations within health care.

**Conclusion:**

The majority found it problematic to assess the level and duration of work incapacity, but also other types of problems like unnecessarily long sick-leave periods due to different types of waiting times. The findings have implications for different kinds of organisational and managerial support and training in sickness certification issues, like guidance to assess the level and duration of work incapacity.

## Background

Sickness absence due to mental diagnoses has increased in Western countries [1,2]. Psychiatrists issue a considerable number and an increasing share of the sickness certificates in Western countries [3,4]. Sickness certification in psychiatric care constitutes an increasing workload, and is particularly complex as it includes a number of new diagnoses for which the criteria are still under discussion [5]. Several new and effective therapies have been introduced with sometimes faster recovery. Increasing teamwork in psychiatric health care may imply that psychiatrists more often are involved as consulting specialists. The possible side effects of being sickness absent with psychiatric diagnoses are more troublesome to handle than in somatic health care [6,7], and several psychiatrists even experience this to be a work environmental problem [8].

In Sweden, after one week of self-certification, all patients need a medical certificate issued by a physician to be considered for sickness benefits. These certificates have great impact on whether the employer or, after two weeks of sick leave, the Social Insurance Office, decides that the patient fulfils the criteria for benefits or not [9]. Consultations regarding sickness certification involve several different tasks such as; deciding whether the patient has a disease or injury, assessing the patient’s function and work capacity, together with the patient consider advantages and disadvantages of being on sick leave, making a decision about the duration and degree of sick leave, cooperating with others when needed, issuing a certificate, and documenting measures taken [10,11]. It is also possible that the views on work capacity in mental illness is even less developed than in other health care areas, as are standards for what generally constitute the normal mental and social work demands in different occupations.

Physicians in general consider sick-listing tasks as problematic [12], especially regarding conflicts with patients, assessment of work incapacity, estimation of length and degree of certification, and prolongation of sick-leave initially certified by another physician [10,13]. One recent study indicated that the odds ratios for finding these tasks problematic were highest in primary health care [10], but that physicians in some other clinical settings, nevertheless, had such consultations more often than GPs and many of them also found these tasks problematic, e.g. in rheumatology, psychiatry, and orthopaedics. Sickness certification is also considered to be an important task for physicians other than general practitioners [13,14]. The physicians in these studies experienced problems with numerous tasks related to sickness certification and the problems varied considerably between types of clinics. In a study with limited participation of psychiatrists, sick-listing was found to be influenced by the physician's specialty and gender, and physicians were strongly influenced by how patients presented their problems [15]. So far, most studies regarding physicians' sickness-certification practices have targeted primary health care and general practitioners [11,16]. Psychiatrists have received little attention, resulting in a lack of knowledge about frequency and specific problems in the sickness certification tasks needed to reach optimal professional practice in this field.

The objective of this study was to explore frequencies of problematic issues in the sickness certification tasks and experiences of severity regarding these problematic issues among psychiatrists.

## Method

Data from a comprehensive questionnaire (see Additional file 1) about physicians’ sick-listing practices sent to all physicians in Sweden were analysed. The study population receiving this questionnaire comprised all the 36,898 physicians who lived and worked in Sweden in October 2008. The questionnaire was developed based on previous studies, in interaction with clinicians and other researchers, and a pilot study [11,13,17]. Information about age, sex, year of medical degree and of registration (after two years of internship), and type of specialist qualifications (after at least five more years of resident training) were obtained mainly from the National Board of Health and Welfare. Three reminders were posted to non-respondents, and the response rate was 60.6%.

The study population consisted of the 1185 physicians who answered the questionnaire and who were specialists in psychiatry (board certified psychiatrists) (20.9% of them also had another speciality). In this study, we restricted the study group to those 723 (61.0%) psychiatrists who were under 65 years of age and were working mainly in psychiatric health care. From among these physicians, we included the 579 physicians (53% women) in the analyses who ‘at least once a week had consultations involving sickness certification’ (Table 1).

**Table 1:** Study population, response rate, number under 65 years of age mainly working in psychiatric health care, and frequency of sickness-certification cases, respectively

	**Responding psychiatrists**	**<65 years of age, and working in psychiatric health care**		Frequency of consultations involving consideration of sickness certification among the responding psychiatrists working in psychiatric health care *								**Is inpatient care part of your daily work?**			
				**At least 6 times per week**		**1-5 times per week**		**A few times per month or year**		**Never or almost never**		**Yes**		**No**	
	**N**	**n**	**%**	**n**	**%**	**n**	**%**	**n**	**%**	**n**	**%**	**n**	**%**	**n**	**%**
All	1185	723	61.0	**393**	55.0	**186**	26.0	68	9.5	68	9.5	284	43.2	374	56.8
Women	599	383	63.9	206	54.4	99	26.1	41	10.8	33	8.7	143	40.6	209	59.4
Men	586	340	58.0	187	55.7	87	25.9	27	8.0	35	10.4	141	46.1	165	53.9
31-54 years	464	336	72.4	202	60.5	76	22.8	35	10.5	21	6.3	170	53.8	146	46.2
55-64 years	518	387	74.7	191	50.1	110	28.9	33	8.7	47	12.3	114	33.3	228	66.7
> 65 years	203	-	-	-	-	-	-	-	-	-	-	-	-	-	-

The study group consisted of the 579 psychiatrists who had such consultations at least once a week (bold figures).

*Missing 8 (1.1%).

**Figure 1:**
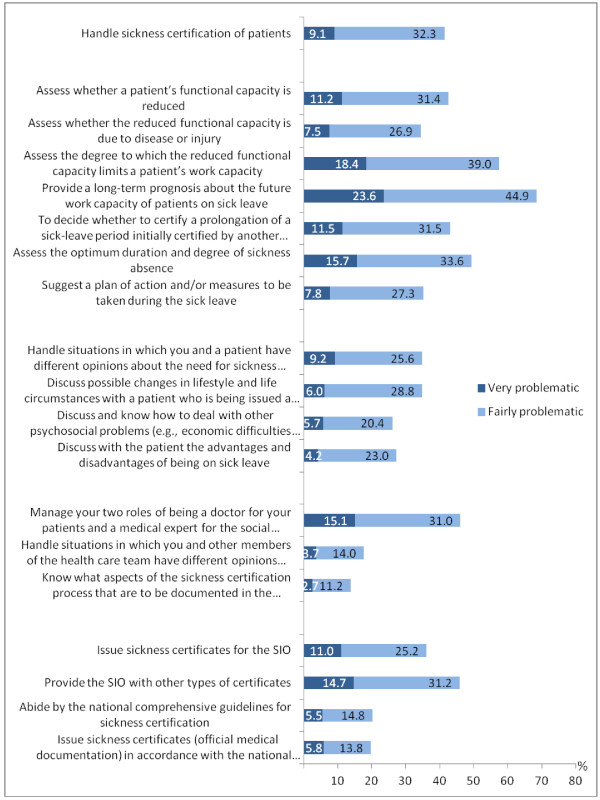
**Percentage of psychiatrists (N=579) rating different aspects of sickness certification as very or fairly problematic.** (SIO is Social Insurance Office).

**Figure 2:**
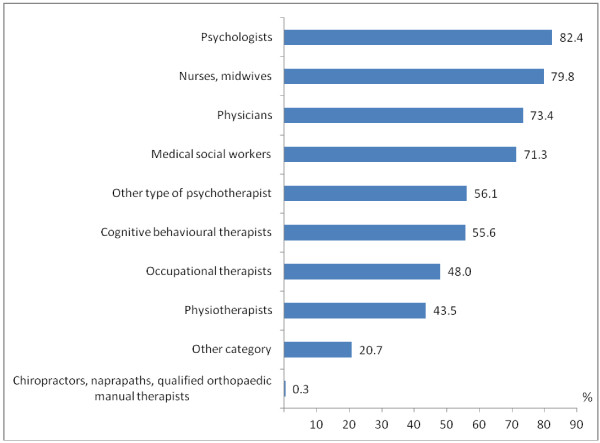
Percentage of psychiatrists (N=579) reporting having access to different professional groups/expertise in their daily work with patients.

**Figure 3:**
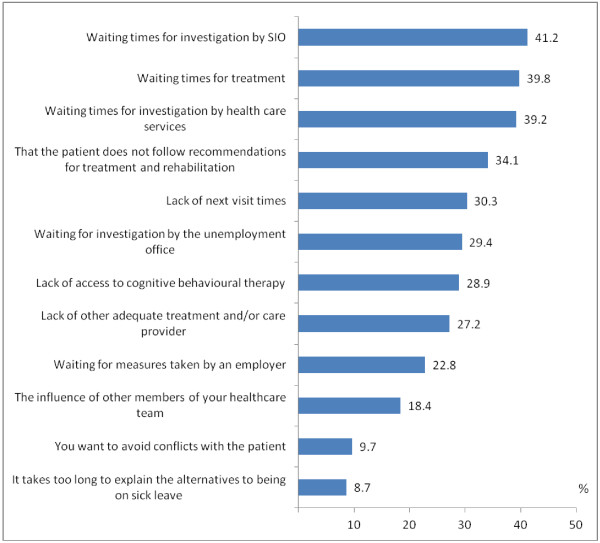
**Percentage of psychiatrists (N=579) who, for different reasons, issued sickness certificates for unnecessarily long periods at least once a month.** (SIO is Social Insurance Office).

Answers to the following types of items were analysed: frequency and severity of problems concerning handling of sickness certifications, access to professional support or expertise, and frequency of issuing certificates for unnecessarily long sick-leave periods. The specific questions (number 6, 8–10, 12–14 and 23 in the questionnaire) and their wording are presented in Table 2 and in Figures 1, 2 and 3.

**Table 2:** Proportion of psychiatrists (N=597) reporting frequencies of different situations regarding sickness certification

**Nr**	**How often in your clinical work do you…**	**At least once a week**	**About once a month/ a few times/year**	**Never or almost never**
1	… find sickness certification cases to be problematic?	11.7	75.6	12.7
2	… encounter a patient who wants to be on sick leave for some reason other than work incapacity due to disease or injury?	4.1	59.3	36.6
3	… say no to a patient who asks for a sickness certificate?	1.4	47.0	51.5
4	… have patients who partly or completely say no to a sick leave you suggest?	1.3	43.7	55.0
5	… issue a sickness certificate so that a patient will be eligible for higher benefits than unemployment or welfare benefits?	.0	31.8	68.2
6	… have conflicts with patients about sickness certification?	3.3	39.6	57.1
7	… worry that a patient will report you to the medical disciplinary board in connection with sickness certification?	4.2	31.6	64.2
8	… feel threatened by a patient in connection with sickness certification?	1.6	28.2	70.2
9	… worry that patients are going to change physician if you don’t issue a sickness certificate?	.0	36.4	63.6
10	… have patients saying they will change physician if you don’t issue a sickness certificate	2.3	13.8	83.8
11	… collaborate with or refer patients to physical or occupational therapists when handling cases involving sickness certification?	4.7	58.8	36.5
12	… collaborate with or refer patients to social workers and/or psychologists when handling cases involving sickness certification?	13.0	66.2	20.8
13	… confer with other doctors when handling cases involving sickness certification?	1.9	39.5	58.6
14	… have time alone or with colleagues for supervision/feedback/reflection related to sickness certification issues?	1.5	40.0	58.5
	**When handling sickness certification tasks, how often do you not have enough time…**			
15	… with your patients?	60.9	22.6	16.5
16	… to manage patient-related aspects (e.g. issuing certificates, contacting other stakeholders, documentation and meetings)?	76.6	17.0	6.3
17	… for further education, supervision or reflection?	69.2	20.2	10.6
	**How often in your clinical work do you…**			
18	… refer/send patients to occupational health services?	.3	30.5	69.2
19	… issue sickness certificates to patients without seeing them (e.g. by telephone)?	4.5	62.0	33.5
20	… apply the new national guidelines for sickness certification?	6.8	55.7	37.5
21	… or does your health care team participate in coordination meetings with social insurance and/or employers about patients to whom you issue sickness certificates?	1.3	66.3	32.4
22	… or does your care team contact employers in ways other than via the coordination meetings?	1.1	45.9	53.0
23	… contact social services when handling cases involving sickness certification?	1.7	46.1	52.3
24	… contact the employment offices when handling cases involving sickness certification?	1.0	37.6	61.4
25	… wish there was someone (e.g. a coach or an advisor) who could coordinate measures implemented for patients?	15.6	54.0	30.5

Response alternatives are presented as numbers and percentages, in terms of either frequency or severity. Some of the items were introduced with the question ‘How often in your clinical work do you …?’ or ‘When handling sickness certification tasks, how often do you …?’ There were six response alternatives from ‘More than 10 times a week’ to ‘Never or almost never’. These were categorized into three groups (Table 2). Other items were introduced with the question ‘How problematic do you generally find it to …?’ with four response alternatives from ‘Very’ to ‘Not at all’ (Figure 1). The third kind of item was introduced with the question ‘How often do you certify unnecessarily long sick-leave periods due to …?’ There were five response alternatives from ‘Every day’ to ‘Never or almost never’ (Figure 3).

The study was approved by the Regional Ethical Review Board of Stockholm.

## Results

Regarding the general question as to how often the participants ‘found handling sickness certification consultations problematic’, 11.7% did so at least once a week and 87.3% did so at least a few times per year (Table 2). At least once a week, 4.1% of the psychiatrists had ‘a patient who wanted a sickness certificate for some reason other than work incapacity due to disease or injury’. Furthermore, 3.3% reported ‘having conflicts with patients about sickness certification’ at least once a week, and 85.9% reported having such conflicts at least a few times per year. About 13.0% reported that they at least once a week collaborated with or referred sick-listed patients to a medical social worker and/or psychologist. When handling sickness certification cases, a majority (60.9%) of the psychiatrists reported ‘not having enough time with the patient’, and 76.6% reported ‘not having enough time to manage patient related aspects’ such as contacting other stakeholders, at least once a week.

Regarding the severity of the reported problems, 41.4% responded to the general question about the handling of sickness certification that certification was very or fairly problematic (Figure 1). Issues that rated highest as very or fairly problematic concerned providing a long-term prognosis about the future work capacity (68.5%) and assessment of a patient’s work capacity (57.4%). Also rated high as very or fairly problematic were to assess the optimum duration and degree (part or full time) of sickness absence (49.3%), issuing other types of certificates for the Social Insurance Office (45.9%), and managing the two involved roles; as the patient’s physician and as a medical expert (45.1%).

A majority of the psychiatrists had access in their daily work to members of several different professions (Figure 2). A majority (51.6%) also had extensive or some support regarding sickness certification cases from their immediate manager (Table 3).

**Table 3:** Proportion of psychiatrists (N=579) who reported having management support and worked at clinic with a workplace policy regarding handling of sickness certification of patients


Do you and your colleagues at your clinic/practice have a workplace policy for handling matters related to sickness certification?	Yes, and it’s well established	Yes, but it’s not well established	No	Don’t know	Not applicable. I don’t work in a clinical unit
	15.2	12.6	56.0	13.4	2.8
Do you have support from your immediate manager at your practice/clinic regarding sickness certification cases?	Yes, extensive support	Yes, some support	No	Not applicable. I don’t have a manager	Not applicable. I don’t work at a clinical unit
	15.6	36.0	35.2	12.9	0.4

At least once a month, more than one third of the psychiatrists certified unnecessarily long sick-leave periods due to waiting times for Social Insurance Office investigations, or for treatments or investigations within the health care and also due to that the patient does not follow recommendations for treatment and rehabilitation (Figure 3).

## Discussion

Psychiatrists’ sickness certification practices were examined in a nationwide survey, which to our knowledge is the first of its kind. A majority (81.0%) had such consultations at least once a week. A large proportion (87.3%) of the psychiatrists experienced sickness certification consultations as problematic at least a few times per year. More than one third certified unnecessarily long sick-leave periods at least once a month due to different types of waiting times.

The experience of severity in these problematic cases varied with response on questions about specific problematic sickness certification issues. The majority found it very or fairly problematic to assess the level of work incapacity and prognosticate the duration of incapacity, had access in their daily work to members of several different professions, and experienced support from immediate management.

Despite considerable differences between countries regarding health care and sickness insurance systems, the problems found in our study are similar to those also reported in other countries [10,11,16]. In a British study, Hussey et al. found that the doctor-patient relationship was perceived to be in conflict with the current role of GPs in sickness certification, and that the GPs experienced contradictory demands from other system stakeholders [18]. Wynne-Jones et al. highlighted the main issues in sickness certification faced by GPs in the United Kingdom [16]. Conflicts with patients and other stakeholders, conflicting role responsibilities, and barriers to good practice were recurrent themes. In Norway, Sheel et al. found lack of information, lack of time, and workflow barriers such as poor communication and poor coordination of activities between stakeholders to the three major such barriers [19]. In a qualitative analysis of responses to open-ended questions in a questionnaire completed by physicians in Sweden, similar types of problems were reported, and many respondents lacked support in handling these problems [20].

Hussey et al. also found that there appeared to be a deliberate misuse of the system by the physicians, that is, GPs, possibly due in part to their problems in handling related conflicts [18]. This is in line with our findings that unnecessarily long sick-leave periods were sometimes issued by the psychiatrists in order to avoid conflicts with patients.

None of the international studies we found has focused on psychiatrists. The studies show similar general problems in the sickness certification task as in the Swedish ones. Hence, the similarities might be the same studying a specialist group as psychiatrists. We look forward to more international studies in this research field.

In our study, only a few of the psychiatrists met a patient at least once a week who wanted to be on sick leave for some reason other than work incapacity due to disease or injury. Even fewer, 1.4% at least once a week said no to a patient who asked for a sick note. This might be due to having developed strategies for handling such requests so that the patient refrained from explicitly asking for a sickness certificate. Nevertheless, at least once a month almost one out of ten issued unnecessarily long sick-leaves to avoid conflicts with patients. This is in line with Englund’s findings that physicians more often issued sickness certificates for patients who actually demanded them than for those who did not do so [15]. Another study, however, found that the physician’s decision to offer a sickness certificate was not influenced by the patient’s demand [21]. Both these studies were based on case vignettes and it is likely that different wording in case vignettes, as well as in questionnaires, could affect the results. There are also findings showing that physicians sometimes act in a way that is contradictory to their own beliefs when actually issuing sickness certificates [22]. On the other hand, almost once a month half of them had patients who refused sick leave that was suggested by the physicians. With reference to the studies we have found it seems to be difficult to generalize our results regarding psychiatrists’ problems in sickness certification tasks to other specialist groups.

A third of the psychiatrists stated that they issued sickness certificates at least once a month for unnecessarily long periods due to waiting times for Social Insurance Office investigations, or for treatments or investigations within health care. Engblom et al. found that ongoing long-term sick-listing cases were perceived as problematic by GPs, and that some of the most commonly reported rehabilitation measures were referrals to psychotherapy and/or physiotherapy, and the prescribing of antidepressants [23]. This could reflect that even though patients have access to rehabilitation, the case remains problematic from the physician’s point of view. Again, the situation may also differ between psychiatrists and GPs, not merely regarding type of medical problems but also severity of psychiatric disorders of patients.

The main strengths of the study are the large number of participants, the fact that all of the psychiatrists in an entire country were included, the relatively high response rate compared to other studies of this type, and the many and detailed questions about different aspects of sickness certification. Nevertheless, the dropout is also a limitation; 41% of the physicians who had specialised in psychiatry did not respond. Also, as in any questionnaire study the results can be a matter of discussion regarding how the participants have interpreted the questions. For instance, their understanding of concepts like ‘problem’ or ‘conflict’ might differ, and reported frequencies do not necessarily correspond to what actually is experienced or done in practice. We were careful not to ask questions about opinions and attitudes, as these are even more difficult to interpret and are seldom associated with actual behaviour such as in sickness certification situations [24,25]. However, we believe that our results are good estimates of the type and severity of problems concerning sickness certification experienced by psychiatrists in psychiatric health care.

Sickness certification needs to be considered as an important task for physicians, with implications for specialist training, continuous education, as well as future inter-professional collaboration in sickness certification. More emphasis on the prerequisites and possibilities for psychiatrists to gain, maintain, and optimally exercise professional competence in insurance medicine, in the context in which they work, is warranted. To gain further knowledge concerning psychiatrists’ sickness certification in clinical practice and concerning how the problems are perceived and handled, observational studies are an option.

## Conclusions

Problems related to sickness certification were frequent and were considered severe among psychiatrists, both in general and regarding specific tasks. The majority found it problematic to assess the level and duration of work incapacity, had access to members of several different professions, and experienced support from immediate management. More than one third frequently certified unnecessarily long sick-leave periods due to different types of waiting times.

The findings have implications for different kinds of organisational and managerial support and training in sickness certification issues, like guidance to assess the level and duration of work incapacity, more of co-operation inter-professional and with stakeholders regarding sickness certification tasks.

## Competing interests

The authors declare that they have no competing interests.

## Authors' contributions

GHN had the main responsibility for design, analysis, and preparation of the manuscript. LK performed the statistical analyses. GHN, BA, CL and KA planned and developed the questionnaire. All authors participated in the analysis and drafted the manuscript. All authors read and approved the final manuscript.

## Key points

- Sickness certification is a common task among psychiatrists.

- A majority of psychiatrists consider handling sickness certification as problematic.

- Psychiatrists frequently certify unnecessarily long sick-leave periods.

## Pre-publication history

The pre-publication history for this paper can be accessed here:

http://www.biomedcentral.com/1472-6963/12/362/prepub

## Supplementary Material

Additional file 1 Questionnaire to physicians about their sickness-certification practices. Click here for file

## References

[B1] Järvisalo J Anderson B Boedeker W Houtman I Mental disorders as a major challenge in prevention of work disability 2005 Helsinki: Kela Volume 66

[B2] Henderson M Glozier N Holland Elliot K Long term sickness absence BMJ 2005 330 9 802 803 10.1136/bmj.330.7495.802 15817531 PMC556060

[B3] Arrelöv B Alexanderson K Hagberg J Löfgren A Nilsson G Ponzer S Dealing with sickness certification - a survey of problems and strategies among general practitioners and orthopaedic surgeons BMC Public Health 2007 7 147 273 17910746 10.1186/1471-2458-7-273 PMC2089078

[B4] Wynne-Jones G Mallen CD Welsh V Dunn KM Rates of sickness certification in European primary care: A systematic review Eur J Gen Pract 2008 14 99 108 10.1080/13814780802687521 19153887

[B5] Frances A It′s not too late to save "normal" Los Angeles Times 2010 March 01

[B6] Vingård E Alexanderson K Norlund A Swedish Council on Technology Assessment in Health Care (SBU). Chapter 9. Consequences of being on sick leave Scandinavian Journal of Public Health 2004 32 Supplemet 63, Review 207 215 10.1080/14034950410021899 15513658

[B7] Floderus F Göransson S Alexanderson K Aronsson G Self-estimated life situation in patients on long-term sick leave J Rehabil Med 2005 37 291 299 10.1080/16501970510034422 16203618

[B8] Ljungquist T Arrelöv B Lindholm C Wilteus AL Nilsson GH Alexanderson K Physicians who experience sickness certification as a work environmental problem: where do they work and what specific problems do they have? A nationwide survey in Sweden BMJ Open 2012 2 2 e000704 10.1136/bmjopen-2011-000704 PMC3293140 22382120

[B9] Söderberg E Alexanderson K Sickness certificates as a basis for decisions regarding entitlement to sickness insurance benefits Scandinavian Journal of Public Health 2005 33 314 320 10.1080/14034940510005798 16087494

[B10] Lindholm C Arrelöv B Nilsson G Löfgren A Hinas E Skåner Y Ekmer A Alexanderson K Sickness-certification practice in different clinical settings; a survey of all physicians in a country BMC Public Health 2010 10 752 10.1186/1471-2458-10-752 21129227 PMC3016384

[B11] Wahlström R Alexanderson K Swedish Council on Technology Assessment in Health Care (SBU). Chapter 11. Physicians' sick-listing practices (review) Scand J Public Health Suppl 2004 63 222 255 15513660 10.1080/14034950410021916

[B12] Alexanderson K Norlund A Eds Sickness absence - causes, consequences, and physicians' sickness certification practice. A systematic literature review by the Swedish Council on Technology Assessment in Health Care Scandinavian Journal of Public Health 2004 32 Supplement 63 1 263 15513647 10.1080/14034950410003826

[B13] Löfgren A Arrelöv B Hagberg J Ponzer S Alexanderson K Frequency and nature of problems associated with sickness certification tasks: a cross sectional questionnaire study of 5455 physicians Scand J Prim Health Care 2007 25 3 178 185 10.1080/02813430701430854 17846937 PMC3379778

[B14] Löfgren A Silén C Alexanderson K How physicians have learned to handle sickness-certification cases Scandinavian Journal of Public Health 2010 Accepted 10.1177/1403494810393301 21262852

[B15] Englund L Tibblin G Svärsudd K Variations in sick-listing practice among male and female physicians of different specialites based on case vignettes Scand J Prim Health Care 2000 1 48 52 10811044 10.1080/02813430050202569

[B16] Wynne-Jones G Mallen C Main C Dunn K What do GPs feel about sickness certification? A systematic search and narrative review Scand J Prim Health Care 2010 28 67 75 10.3109/02813431003696189 20334576 PMC3442320

[B17] von Knorring M Sundberg L Löfgren A Alexanderson K Problems in sickness certification of patients: a qualitative study on views of 26 physicians in Sweden Scand J Prim Health Care 2008 26 1 22 28 10.1080/02813430701747695 18297559 PMC3406623

[B18] Hussey S Hoddinott P Wilson P Dowell J Barbour R Sickness certification system in the United Kingdom: qualitative study of views of general practitioners in Scotland Bmj 2004 328 7431 88 10.1136/bmj.37949.656389.EE 14691065 PMC314050

[B19] Scheel IB Hagen KB Oxman AD Active sick leave for patients with back pain: all the players onside, but still no action Spine 2002 27 6 654 659 10.1097/00007632-200203150-00016 11884914

[B20] Gerner U Alexanderson K Issuing sickness certificates: a difficult task for physicians: a qualitative analysis of written statements in a Swedish survey Scand J Public Health 2009 37 1 57 63 19039091 10.1177/1403494808097170

[B21] Campbell A Ogden J Why do doctors issue sick notes? An experimental questionnaire study in primary care Fam Pract 2006 23 1 125 130 16308327 10.1093/fampra/cmi099

[B22] Englund L Svärdsudd K Sick-listing habits among general practitioners in a Swedish county Scand J Prim Health Care 2000 18 2 81 86 10.1080/028134300750018954 10944061

[B23] Engblom M Alexanderson K Rudebeck CE Characteristics of sick-listing cases that physicians consider problematic–analyses of written case reports Scand J Prim Health Care 2009 27 4 250 255 10.3109/02813430903286286 19958066 PMC3413918

[B24] Gulbrandsen P Hofoss D Nylenna M Saltyte-Benth J Aasland OG General practitioners' relationship to sickness certification Scand J Prim Health Care 2007 25 1 20 26 10.1080/02813430600879680 17354155 PMC3389448

[B25] Watson PJ Bowey J Purcell-Jones G Gales T General practitioner sickness absence certification for low back pain is not directly associated with beliefs about back pain Eur J Pain 2008 12 3 314 320 10.1016/j.ejpain.2007.06.002 17659991

