# Supplementary material for: Psychiatrists′ work with sickness certification: frequency, experiences and severity of the certification tasks in a national survey in Sweden
Source: BMC Health Serv Res. 2012 Oct 17;12:362. doi: 10.1186/1472-6963-12-362 (PMC3480832; doi:10.1186/1472-6963-12-362)
Supplement: Additional file 1 — Questionnaire to physicians about their sickness-certification practices. [file 1472-6963-12-362-S1.pdf]

Questionnaire to physicians about their sickness-certification practices

Karolinska Institutetet, 2008

Contact:
Kristina Alexanderson
Professor, Head of section
Division of Insurance Medicine
Karolinska Institutet
SE-171 77 Stockholm
Sweden

Tel: + 46 8 524 832 00
Fax: + 46 8 524 832 05
Mobile: + 46 702 162 099
E-mail: kristina.alexanderson@ki.se

Copyright © K Alexanderson, Division of Insurance Medicine,
Department of Clinical Neuroscience, Karolinska Institutet, Sweden, 2008.

1. What is your highest level of medical education?

- ☐ Medical degree ⇒ *Go to question 3*
- ☐ Registered physician
- ☐ In resident training to become a specialist
- ☐ Board certified specialist

2. What specfialist training are you currently undergoing?

- ☐ **None**
- ☐ General practice
- ☐ Anaesthesia and intensive care
- ☐ Child and adolescent (medicine, surgery, or psychiatry)
- ☐ Imaging and functional medicine
- ☐ Occupational health
- ☐ Geriatrics
- ☐ Dermatology and sexually transmitted diseases
- ☐ Infectious diseases
- ☐ Internal medicine
- ☐ Surgery
- ☐ Laboratory medicine
- ☐ Neurology
- ☐ Obstetrics/Gynaecology
- ☐ Oncology
- ☐ Orthopaedics
- ☐ Psychiatry/Addiction medicine
- ☐ Rehabilitation medicine
- ☐ Rheumatology
- ☐ Ophthalmic diseases
- ☐ Ear, nose, and throat disorders
- ☐ Other

3. How long have you worked at your current place of work?

- ☐ < 5 years
- ☐ 5–9 years
- ☐ 10 years or more

Copyright © K Alexanderson, Division of Insurance Medicine,
Department of Clinical Neuroscience, Karolinska Institutet, Sweden, 2008.

4. At what type of clinic/practice do you mainly work?

Choose only one of the following options.

- ☐ Child and adolescent (medicine, surgery, or psychiatry)
- ☐ Occupational health
- ☐ Social insurance office/Insurance company
- ☐ Geriatrics
- ☐ Dermatology
- ☐ Infectious diseases
- ☐ Internal medicine
- ☐ Surgery
- ☐ Neurology
- ☐ Gynaecology/Obstetrics
- ☐ Oncology
- ☐ Orthopaedics
- ☐ Psychiatry
- ☐ Rehabilitation
- ☐ Rheumatology
- ☐ Pain management
- ☐ Primary Health Care Centre/Family medicine
- ☐ Ophthalmology
- ☐ Ear, nose, and throat
- ☐ Other type of clinic/practice
  - ☐ Administration, research, or teaching
  - ☐ None

5. In what county or region is your primary place of employment?

☐ Blekinge	☐ Jönköping	☐ Stockholm	☐ Västernorrland
☐ Dalarna	☐ Kalmar	☐ Sörmland	☐ Västmanland
☐ Gotland	☐ Kronoberg	☐ Uppsala	☐ Västra Götaland
☐ Gävleborg	☐ Norrbotten	☐ Värmland	☐ Örebro
☐ Halland	☐ Skåne	☐ Västerbotten	☐ Östergötland
☐ Jämtland			

☐ Other country

6. Does your clinical unit have a joint policy for handling matters related to sickness certification tasks?

- ☐ Yes, and it is well established
- ☐ Yes, but it is not well established
- ☐ No
- ☐ I don’t know
  - ☐ Not applicable, I don’t work in a clinical unit

Copyright © K Alexanderson, Division of Insurance Medicine,
Department of Clinical Neuroscience, Karolinska Institutet, Sweden, 2008.

7. In your opinion, does the county council make efforts to improve the way that the health services handle patients' sickness certification?

☐ Yes, definitely
☐ Yes, to some extent
☐ No, not at all

} The effort is sufficient ☐ Yes ☐ No

8. How often in your daily clinical work do you have consultations including consideration of sickness certification?

More than 20 times a week	6–20 times a week	1–5 times a week	About once a month	A few times a year	Never or almost never
☐	☐	☐	☐	☐	☐

If you chose the response "Never or almost never", this was your final question. Thank you for your participation!

9. Is inpatient care part of your daily work?

☐ Yes
☐ No

10. What professional groups/expertise do you have access to in your daily work with patients?

☐ Occupational therapists
☐ Social workers
☐ Physicians
☐ Psychologists
☐ Physiotherapists
☐ Nurses, midwives
☐ Cognitive behavioural therapists
☐ Other type of psychotherapist
☐ Chiropractors, naprapaths, qualified orthopaedic manual therapists
☐ Other category

**11. When handling sickness certification tasks, how often do you lack time...**	Every day	About once a week	About once a month	A few times a year	Never or almost never
...with your patients?	☐	☐	☐	☐	☐
...to manage patient-related aspects (e.g., issue certificates, contact stakeholders, documentation, and meetings)?	☐	☐	☐	☐	☐
...for further education, supervision, or reflection?	☐	☐	☐	☐	☐

Copyright © K Alexanderson, Division of Insurance Medicine,
Department of Clinical Neuroscience, Karolinska Institutet, Sweden, 2008.

12.	How often in your clinical work do...	More than 10 times a week	6–10 times a week	1–5 times a week	About once a month	A few times a year	Never or almost never
	...you find it problematic to handle sickness certification?	☐	☐	☐	☐	☐	☐
	...you encounter a patient who wants to be on sick leave for some reason other than work incapacity due to disease or injury?	☐	☐	☐	☐	☐	☐
	...patients partly or completely say no to sick leave you suggest?	☐	☐	☐	☐	☐	☐
	...you say no to a patient who wants a sickness certificate?	☐	☐	☐	☐	☐	☐
	...you refer/send patients to occupational health services?	☐	☐	☐	☐	☐	☐
	...you issue a sickness certificate so that a patient will be eligible for higher benefit than unemployment or welfare benefits?	☐	☐	☐	☐	☐	☐
	...you experience conflicts with patients about sickness certification?	☐	☐	☐	☐	☐	☐
	...you worry that a patient will report you, to e.g. a disciplinary board, regarding sickness certification?	☐	☐	☐	☐	☐	☐
	...you feel threatened by a patient in connection with sickness certification?	☐	☐	☐	☐	☐	☐
	...you issue sickness certificates to patients without seeing them (e.g., by telephone)?	☐	☐	☐	☐	☐	☐
	...you worry that patients will go to another physician if you don’t issue a sickness certificate?	☐	☐	☐	☐	☐	☐
	...patients say that they will change physician if you don’t issue a sickness certificate?	☐	☐	☐	☐	☐	☐
	...you apply the newly introduced national sickness certification guidelines?	☐	☐	☐	☐	☐	☐
	...you have time scheduled, alone or with colleagues, for supervision/feedback/reflection regarding sickness certification issues?	☐	☐	☐	☐	☐	☐
	...you or your health care team participate in coordination meetings with social insurance and/or employer regarding sickness certified patients?	☐	☐	☐	☐	☐	☐
	...you or your care team have contact with employers in ways other than via the coordination meetings?	☐	☐	☐	☐	☐	☐
	...you collaborate with or refer patients to social workers and/or psychologists in sickness-certification cases?	☐	☐	☐	☐	☐	☐

Copyright © K Alexanderson, Division of Insurance Medicine,
Department of Clinical Neuroscience, Karolinska Institutet, Sweden, 2008.

How often in your clinical work do...	More than 10 times a week	6–10 times a week	1–5 times a week	About once a month	A few times a year	Never or almost never
...you collaborate with or refer patients to physical or occupational therapists in sickness-certification cases?	☐	☐	☐	☐	☐	☐
...you confer with other physicians when handling cases involving sickness certification?	☐	☐	☐	☐	☐	☐
...you have contact with social services regarding sickness-certification cases?	☐	☐	☐	☐	☐	☐
...you have contact with the employment offices regarding sickness-certification cases?	☐	☐	☐	☐	☐	☐
...you lack for someone (e.g., a coach or case manager) who coordinates measures for the patients?	☐	☐	☐	☐	☐	☐

**13.** **How often do you certify unnecessarily long sick-leave periods due to ...**	Every day	About once a week	About once a month	A few times a year	Never or almost never
...lack of next visit times?	☐	☐	☐	☐	☐
...waiting times for investigation by health care services?	☐	☐	☐	☐	☐
...waiting for investigation by the social insurance office (SIO)?	☐	☐	☐	☐	☐
...waiting for investigation by the unemployment office?	☐	☐	☐	☐	☐
...waiting for measures taken by an employer?	☐	☐	☐	☐	☐
...waiting times for treatment?	☐	☐	☐	☐	☐
...lack of access to cognitive behavioural therapy?	☐	☐	☐	☐	☐
...lack of other adequate treatment and/or care provider? *(Comments can be given on page 12)*	☐	☐	☐	☐	☐
...that the patient does not follow recommendations for treatment and rehabilitation?	☐	☐	☐	☐	☐
...you want to avoid conflicts with the patient?	☐	☐	☐	☐	☐
...it takes too long to explain the alternatives to being on sick leave?	☐	☐	☐	☐	☐
...the influence of other members of your healthcare team?	☐	☐	☐	☐	☐

Copyright © K Alexanderson, Division of Insurance Medicine,
Department of Clinical Neuroscience, Karolinska Institutet, Sweden, 2008.

**14.**	**How problematic do you generally find it to...**		Very	Fairly	Some-what	Not at all
	...handle sickness certification of patients?		☐	☐	☐	☐
	...assess whether a patient's functioning is reduced?		☐	☐	☐	☐
	...assess whether the reduced functioning is due to disease or injury?		☐	☐	☐	☐
	...assess the degree to which the reduced functioning limits a patient's work capacity?		☐	☐	☐	☐
	...discuss with the patient the advantages and disadvantages of being on sick leave?		☐	☐	☐	☐
	...suggest a plan of action and/or measures to be taken during the sick leave?		☐	☐	☐	☐
	...provide a long-term prognosis about the future work capacity of patients on sick leave?		☐	☐	☐	☐
	...manage the two roles as the patient's treating physician and as a medical expert for the social insurance office and other authorities?		☐	☐	☐	☐
	...discuss possible lifestyle and life situation changes with a patient that you sickness certify?		☐	☐	☐	☐
	...discuss and know how to deal with other psychosocial problems (e.g., economic difficulties or physical or substance abuse) when handling a patient on sick leave?		☐	☐	☐	☐
	...know what aspects of the sickness-certification process to be documented in the patient's medical file?		☐	☐	☐	☐
	...handle prolongation of a sick-leave period initially certified by another physician?		☐	☐	☐	☐
	...assess the optimum duration and degree (part or full time) of sickness absence?		☐	☐	☐	☐
	...handle situations in which you and a patient have different opinions about the need for sick leave?		☐	☐	☐	☐
	...write sickness certificates for the Social Insurance Office?		☐	☐	☐	☐
	...write other certificates for the Social Insurance Office?		☐	☐	☐	☐
	...adhere to the national overarching sickness certification guidelines?	Have not used those guidelines ☐	☐	☐	☐	☐
	...issue sickness certificates in accordance with the national diagnosis-specific sickness certification guidelines?	Have not used those guidelines ☐	☐	☐	☐	☐
	...handle situations in which you and other members of the health care team have different opinions about sickness certifying a patient?	Not applicable ☐	☐	☐	☐	☐

Copyright © K Alexanderson, Division of Insurance Medicine,
Department of Clinical Neuroscience, Karolinska Institutet, Sweden, 2008.

15. **In general, how satisfied are you regarding your contacts with the social insurance office (SIO)?**

☐ Very satisfied
☐ Somewhat satisfied
☐ Somewhat dissatisfied
☐ Very dissatisfied
☐ Have no contacts with the SIO

How often do you usually have contact with SIO staff concerning sickness certification cases?

Do not include writing sickness certificates

☐ Daily
☐ Once or a few times a week
☐ About once a month
☐ A few times a year
☐ Never or almost never

Do you have regular, scheduled contacts with the SIO? *(e.g., coordinating meetings, rehabilitation meetings, or SIO officers at your practice)*

☐ Yes, to a satisfactory degree
☐ Yes, but you would prefer more extensive contact
☐ No, but you would like to
☐ No, not needed

How easy/difficult do you find it is to get in touch with SIO case officers?

☐ Not relevant
☐ Very easy
☐ Fairly easy
☐ Neither easy nor difficult
☐ Fairy difficult
☐ Very difficult

Copyright © K Alexanderson, Division of Insurance Medicine,
Department of Clinical Neuroscience, Karolinska Institutet, Sweden, 2008.

16. What difficulties, if any, do you experience in your contacts with the Social Insurance Office (SIO)?

Choose one or more options.

- ☐ no such difficulties
- ☐ It takes too much time
- ☐ It's difficult to find out who to talk to
- ☐ It's difficult to get through by phone
- ☐ The SIO is closed when you have time for contact
- ☐ The SIO staff do not contact you, even if you ask them to
- ☐ The SIO doesn't coordinate rehabilitation measures
- ☐ The SIO doesn't contact the patients
- ☐ You encounter a negative attitude from SIO
- ☐ You seem to speak different languages
- ☐ Ambiguities concerning matters of confidentiality
- ☐ Patients are often assigned new SIO case officers (lack of continuity)
- ☐ SIO case officers question you/your assessments
- ☐ SIO medical officers question you/your assessments
- ☐ It's difficult to convey information through the pre-printed SIO forms for medical certificates
- ☐ Different interpretations of the new national sickness certification guidelines
- ☐ The patient is negative towards you contacting the SIO
- ☐ Other problems *(Please give comments on p. 12)*

17. Considering your future contacts with the SIO, how important are the following options?

You will not be in contact with the SIO ☐ ⇒ *Go to question 18*

	Very important	Fairly important	Not important at all
More coordinating meetings (e.g., with the SIO, the employer, or the employment office, and the patient)	☐	☐	☐
Fewer coordinating meetings	☐	☐	☐
General dialogue about sickness certification and insurance medicine	☐	☐	☐
Regular, scheduled meetings at the SIO to discuss patients	☐	☐	☐
Regular, scheduled meetings at your practice	☐	☐	☐
Contact by e-mail and/or the Internet (possibly encrypted)	☐	☐	☐
Contact by ordinary mail	☐	☐	☐
Contact by telephone	☐	☐	☐
A contact person that you can quickly reach when necessary	☐	☐	☐
SIO case officers at your practice	☐	☐	☐
To be able to quickly obtain information from the SIO about the patient's previous sick leave	☐	☐	☐
To be able to meet a patient together with relevant SIO officer early in the handling of a case	☐	☐	☐

Copyright © K Alexanderson, Division of Insurance Medicine,
Department of Clinical Neuroscience, Karolinska Institutet, Sweden, 2008.

To get information from the SIO about their handling and decisions regarding patients you have issued certificate for	☐	☐	☐

18. Do the new national sickness certification guidelines provided by the National Board of Health and Welfare facilitate your contacts with...

	Yes	No
… healthcare staff?	☐	☐
… patient's workplace or employment office?	☐	☐
… your patient?	☐	☐
… the SIO?	☐	☐

☐ I don't use the indicated guidelines

Please, on page 12 give your views give your views on whether and how the national sickness certification guidelines need to be further developed!

**19. To what extent have the following helped you to develop your competence in handling sickness-certification cases?**	To a large extent	To a fairly large extent	To some extent	Not at all	Not relevant
Undergraduate studies	☐	☐	☐	☐	
Internship	☐	☐	☐	☐	☐
Resident training	☐	☐	☐	☐	☐
Further training arranged by the county council	☐	☐	☐	☐	☐
Education/training opportunities other than those listed above	☐	☐	☐	☐	☐
Your own searches for information (e.g., in the literature or the Internet)	☐	☐	☐	☐	
Contacts with other physicians and/or other healthcare staff	☐	☐	☐	☐	
Contacts with patients	☐	☐	☐	☐	
Contacts with Social Insurance Office staff	☐	☐	☐	☐	☐
Training arranged by the Social Insurance Office	☐	☐	☐	☐	☐
Contacts with employers	☐	☐	☐	☐	☐
Information via media	☐	☐	☐	☐	
Targeted information efforts (e.g., made by a regional sickness certification committee)	☐	☐	☐	☐	☐
Teaching and supervising others about these issues	☐	☐	☐	☐	☐
Conferences, seminars	☐	☐	☐	☐	☐
The newly introduced national sickness certification guidelines	☐	☐	☐	☐	

Copyright © K Alexanderson, Division of Insurance Medicine,
Department of Clinical Neuroscience, Karolinska Institutet, Sweden, 2008.

Copyright © K Alexanderson, Division of Insurance Medicine,
Department of Clinical Neuroscience, Karolinska Institutet, Sweden, 2008.

20.	To what extent do you need to further develop your competence in relation to the following?	To a large extent	To a fairly large extent	To some extent	Not at all
	Assessing patients’ functioning	☐	☐	☐	☐
	Assessing patients’ work capacity	☐	☐	☐	☐
	The demands made on employees in different occupations and/or workplaces	☐	☐	☐	☐
	Assessing the optimum length and degree of sickness absence	☐	☐	☐	☐
	Handling conflicts with patients about the need for sickness certification	☐	☐	☐	☐
	Writing sickness certificates	☐	☐	☐	☐
	Designing optimum plans of action	☐	☐	☐	☐
	Deciding when there is a need to contact the Social Insurance Office	☐	☐	☐	☐
	Sickness insurance rules and laws	☐	☐	☐	☐
	Other types of compensation in the social insurance system (e.g., rehabilitation allowance, disablement benefit, disability pension)	☐	☐	☐	☐
	Private and supplementary insurances that patients often have	☐	☐	☐	☐
	Your options and responsibilities as a physician in sickness certification cases	☐	☐	☐	☐
	The options and responsibilities of the Social Insurance Office in sickness certification cases	☐	☐	☐	☐
	Employers’ options and responsibilities in sickness certification cases	☐	☐	☐	☐
	The options and responsibilities of the Employment Office in sickness certification cases	☐	☐	☐	☐
	Using the new national sickness certification guidelines	☐	☐	☐	☐
	Gender aspects of sickness certification	☐	☐	☐	☐
	Other aspects of diversity that can be related to sickness certification	☐	☐	☐	☐
	The various possibilities in society regarding handling psychosocial problems	☐	☐	☐	☐

Copyright © K Alexanderson, Division of Insurance Medicine,
Department of Clinical Neuroscience, Karolinska Institutet, Sweden, 2008.

21. How do you value the following options with regard to ensuring high quality of your handling of sickness certification cases?

	Very beneficial	Moderately beneficial	Not beneficial
Contacts with fellow physicians and/or other healthcare staff	☐	☐	☐
Contacts with patients' employers and visits to workplaces	☐	☐	☐
Supervision/being tutored	☐	☐	☐
Collaborative or coordinating meetings	☐	☐	☐
Contacts with medical officers at the Social Insurance Office	☐	☐	☐
Contacts with case officers at the Social Insurance Office	☐	☐	☐
The national diagnosis-specific sickness certification guidelines	☐	☐	☐
A joint instrument/protocol for assessment of work capacity	☐	☐	☐
Written information (e.g., books, brochures, articles)	☐	☐	☐
Information from the Internet	☐	☐	☐
Courses in insurance medicine	☐	☐	☐
Courses in conflict management	☐	☐	☐
Conferences, seminars	☐	☐	☐
Having opportunities to contact experts in insurance medicine	☐	☐	☐
Getting a second opinion from other physician regarding a patient	☐	☐	☐
Getting statistics on your issued sickness certificates over the past year	☐	☐	☐
Getting statistics on issued sickness certificates at your unit/practice over the past year	☐	☐	☐
Better information to the general public about the sickness insurance system	☐	☐	☐
Receiving higher remuneration for issuing sickness certificates	☐	☐	☐
A forum in which you can reflect on your work with sickness certification	☐	☐	☐

22. Do sickness certification tasks constitute a work environment problem for you?

☐ Yes, to a great extent
☐ Yes, to some extent
☐ No

23. Do you have support from your immediate manager at your practice/clinic regarding sickness certification cases?

☐ Yes, substantial support
☐ Yes, some support
☐ No
☐ Not applicable, don't have a manager
☐ Not applicable, don't work at a clinical unit

Copyright © K Alexanderson, Division of Insurance Medicine,
Department of Clinical Neuroscience, Karolinska Institutet, Sweden, 2008.

Thank you very much for your participation!

Space for your own comments:

Copyright © K Alexanderson, Division of Insurance Medicine,
Department of Clinical Neuroscience, Karolinska Institutet, Sweden, 2008.
